# The Temporal Dynamic Relationship Between Attention and Crowding: Electrophysiological Evidence From an Event-Related Potential Study

**DOI:** 10.3389/fnins.2018.00844

**Published:** 2018-11-22

**Authors:** Chunhua Peng, Chunmei Hu, Youguo Chen

**Affiliations:** ^1^Laboratory of Emotion and Mental Health, Chongqing University of Arts and Sciences, Chongqing, China; ^2^Collaborative Innovation Center for Brain Science, Chongqing, China; ^3^Key Laboratory of Cognition and Personality (Ministry of Education), Center of Studies for Psychology and Social Development, Faculty of Psychology, Southwest University, Chongqing, China

**Keywords:** crowding, attention, Gestalt grouping, event-related potentials, temporal dynamic

## Abstract

Visual crowding is the difficulty experienced in identifying a target flanked by other objects within the peripheral visual field. Despite extensive research conducted on this topic, the precise relationship between attention and crowding is still debatable. One perspective suggests that crowding is a bottom-up and pre-attentive process, while another suggests that crowding is top-down and attentional. A third perspective proposes that crowding is a combination of bottom-up and top-down processes. To address this debate, the current study manipulated the attention and distance between targets and flankers, while simultaneously measuring event-related potentials, in human participants. Results indicated that, compared to uncrowded targets, crowded targets elicited more negative frontal N1 and P2 activity and a less negative occipital N1 activity, regardless of whether targets were attended or unattended, and a more positive occipital P2 activity when they were attended. Furthermore, the crowded minus uncrowded difference amplitude was more negative over the frontal region and more positive over the occipital region when the targets were attended, compared to when they were unattended during the N1 and P2 stages. This suggests that crowding, a concept that originates from Gestalt grouping, occurs automatically and can be modulated by attention.

## Introduction

The crowding effect is a visual phenomenon in which objects are easily identified in isolation, but become more difficult to identify when surrounded by other objects in the peripheral visual field (Pelli and Tillman, 2008). In a typical scenario, a letter can be identified when it is presented alone, but it cannot be recognized when it is flanked by other letters (Pelli et al., 2004). In addition to English letters, the identification of various other targets has been found to deteriorate in the presence of neighboring objects, including orientation signals (van den Berg et al., 2010), Chinese characters (Yeh et al., 2012; Peng et al., 2013; Zhou et al., 2016), and faces (Farzin et al., 2009).

The relationship between attention and crowding has been the focus of several recent studies. One perspective suggests that crowding is bottom-up and pre-attentive. This assertion is based on the notion that crowding occurs at a lower visual processing level. An early view suggested that the neural activity caused by flanking objects decreases neural activity related to the target due to lateral inhibition (Westheimer and Hauske, 1975). Another view, based on spatial pooling, posits that features from both targets and flankers are pooled, and that these features are compulsorily averaged (Parkes et al., 2001) or combined into a jumbled percept (Levi, 2008; Pelli and Tillman, 2008). Consistent with the pre-attentive account of crowding, Põder (2006, 2007) demonstrated the important role of bottom-up salience in the binding of visual features, which determines the observed extent of crowding. Dakin et al. (2009) indicated that crowding does not reflect an attentional limit, and that crowding and attention rely on distinct neural mechanisms. Specifically, crowding persists even when people are completely unaware of the flankers, which suggests that conscious awareness and attention are not prerequisites for crowding (Ho and Cheung, 2011). Furthermore, Yong et al. (2014) examined the question of why individuals with posterior cortical atrophy (PCA) show excessive crowding in central vision and suggested that crowding in PCA can be regarded as a pre-attentive process that uses averaging to regularize the pathologically noisy representation of letter feature positions.

Another perspective of this topic suggests that crowding is top-down and attentional. This is based upon the idea that crowding occurs at a higher processing level. Therefore, while crowded targets can be perceived, the coarse attentional resolution in peripheral vision limits access of crowded targets to the consciousness (He et al., 1996, 1997). A probabilistic substitution model assumes that crowding results from binding a target and nearby distractors to incorrect spatial locations (Ester et al., 2014, 2015). This perspective has been supported by several studies. Attention improves performance at peripheral locations by enhancing spatial resolution (Yeshurun and Carrasco, 1998). Furthermore, attention reduces the critical target–flanker distance at which the flankers no longer interfere with target identification (Yeshurun and Rashal, 2010). Moreover, attention can be directly guided to various flankers. In a study examining this, attended flankers produced typical lateral interactions, while ignored flankers did not (Freeman et al., 2001). Attention modulates target–flanker integration, rather than just the processing of local flanker elements (Freeman et al., 2003). However, strong and specific attentional modulation of contour–integration mechanisms in early vision are sensitive to collinear configurations (Freeman et al., 2004). Therefore, covert attention to stimuli can increase the weights of their pooled features during crowding (Mareschal et al., 2010).

Electrophysiological studies suggest that attention plays a critical role in crowding. For instance, evidence from an attention-related N2pc component showed that attention functions to minimize interference from flankers at intermediate target–flanker distances (Hilimire et al., 2009, 2010; Bacigalupo and Luck, 2015). Additionally, evidence from a sustained posterior contralateral negativity (SPCN) study showed that working memory may be recruited when attention fails to select the target at small target–flanker distances (Bacigalupo and Luck, 2015). The earliest ERP component, C1, which originates from V1 areas, is suppressed by crowded targets, whereas no suppression of C1 is found if the crowded targets are not attended. This indicates that attention-dependent V1 suppression contributes to crowding at a very early stage of visual processing (Chen et al., 2014). Chicherov et al. (2014) suggested that the P1 component reflects basic stimulus characteristics (i.e., flanker length), and N1 suppression reflects the occurrence of crowding when targets and flankers are grouped into wholes.

A third perspective suggests that crowding is a combination of bottom-up and top-down processes (i.e., crowding occurs automatically and can be modulated by attention). This perspective comes from the Gestalt grouping hypothesis of crowding. Increasing evidence shows that Gestalt grouping is critical for crowding (Malania et al., 2007; Saarela et al., 2009; Sayim et al., 2010). These findings are well explained by the hypothesis that crowding is strong when the flankers are grouped with the target and weaker when the target is segregated from the flankers (Manassi et al., 2012; Herzog et al., 2015; Herzog and Manassi, 2015). For instance, a cortical neural network model has been proposed that uses perceptual grouping and a novel segmentation process to account for several properties of visual crowding, such as effects of flanker length, the number of flanker lines, Gestalt effects, uncrowding effects, and similarity effects (Francis et al., 2017). While the relationship between Gestalt grouping and attention is well known, it is important to note that Gestalt grouping occurs automatically. Thus, visual stimuli that is irrelevant to a given task can be grouped without attention (Russell and Driver, 2005; Lamy et al., 2006), and the formation of visual object representations by grouping can occur outside the focus of voluntary attention (Müller et al., 2010). Electrophysiological evidence has shown that Gestalt stimuli automatically capture attention (Marini and Marzi, 2016). Further, Gestalt grouping has been shown to be modulated by attention. For example, grouping can be modulated by task relevance and attention as early as 100 ms after onset of sensory stimulation (Han et al., 2005), with this interaction between attention and grouping taking place as early in the perceptual process as the primary visual cortex (Wu et al., 2005; Khoe et al., 2006).

The relationship between attention and crowding needs further elucidation. First, additional evidence is necessary to test whether crowding can occur automatically. Behavior studies have shown that crowding is distinct from attention (Dakin et al., 2009) and occurs automatically (Ho and Cheung, 2011; Yong et al., 2014). Yet, several studies have emphasized that attention plays a crucial role in crowding, and that crowding may not occur completely automatically (Herzog et al., 2015; Francis et al., 2017). While N1 components have been found to be suppressed when observers discriminate crowded targets (Chicherov et al., 2014; Ronconi et al., 2016), no study to date has reported whether the N1 signal can be suppressed by crowded targets if they are unattended. Additionally, more direct evidence needs to be provided for the attentional modulation of crowding. Chicherov et al. (2014) reported that N1 suppression was much stronger when the task was to discriminate the crowded Vernier as compared with the flankers length discrimination task. In this task, targets and flankers were in very close proximity, especially for crowded targets; thus, the Vernier may be attended to a certain extent, and this may lead to a slight N1 suppression in the length discrimination task. In the ideal situation, it is necessary to examine the N1 suppression elicited by crowded targets both when the targets are attended and when they are not.

The present study focused on the temporal dynamic relationship between crowding and attention. We combined a crowding paradigm (Yeh et al., 2012; Peng et al., 2013; Zhou et al., 2016) with a cross-modal delayed response oddball paradigm (Wei et al., 2002; Chen et al., 2010). The intermodal selective attention paradigm has been shown to effectively manipulate attention (Alho, 1992; Woods et al., 1992). The cross-modal delayed response paradigm can effectively control attention and minimize target effects (Wei et al., 2002). A series of auditory and visual stimuli are presented in sequence. The presentation of an attended visual stimulus (e.g., a crowded or uncrowded target) is followed by zero, one, or two unattended auditory stimuli (e.g., a tone) and a response signal. This order can also be reversed (e.g., the tone is the attended stimulus, and the crowded or uncrowded target is the unattended stimulus). Participants are required to identify signals of the attended modality, ignore those of the unattended modality, and wait to respond until presentation of the response signal.

Given that crowding is reflected by N1 suppression (Chicherov et al., 2014; Ronconi et al., 2016), three hypotheses on the relationship between attention and crowding can be tested. First, if crowding is bottom-up and pre-attentive, N1 should be suppressed by crowded targets when the targets are not unattended. Next, if crowding is top-down and attentional, N1 suppression would be stronger when the crowded targets are attended than when unattended. Finally, if crowding is a combination of bottom-up and top-down processes, the above two predictions would be observed simultaneously.

Further, additional evidence can be provided to test the Gestalt grouping hypothesis by a measurement of the P2 component. A study reported that P2 engages in grouping elements into a unitary object (Flevaris et al., 2013). If crowding originates from Gestalt grouping, similar results to the N1 stage would be observed during the P2 stage. In other words, a significant difference in P2 amplitude between crowded and uncrowded targets, both in the attended and unattended conditions, will be observed, and the crowded minus uncrowded difference amplitude will be modulated by attention.

## Materials and Methods

### Participants

Eighteen right-handed undergraduate students (two males, 19–24 years of age) participated in this experiment. One participant (female, 23 years of age) was excluded because of excessive eye movement and blinking during the experiment. Participants were not taking any medications and did not suffer from any central nervous system abnormalities or injuries. All were naive to the purpose of the experiment. The study was approved by the institutional review board of Southwest University. Written informed consent was obtained from each participant. The experimental procedure was conducted in accordance with the Declaration of Helsinki (World Medical Association, 2013).

### Experimental Material and Apparatus

Visual stimuli were 20 Chinese single-character words, four Chinese pseudo-characters, a fixation, and a visual response signal. Ten Chinese words indicated animals (e.g., 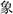 means elephant), and ten indicated inanimate objects (e.g., 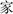 means home). Four pseudo-characters were made from stroke features using TrueType software. They were white, single-bodied, and had no semantic meaning (Peng et al., 2013). The visual angles of all the characters were 1 × 1°. The fixation point was a white dot with a diameter of 0.4°. The visual response signal was a red square with a width of 0.5°. Visual stimuli were presented on a 22-inch Iiyama MA203DT D color monitor with a background screen color of medium gray (RGB color coordinates: 128, 128, 128). The refresh rate of the computer monitor was 85 Hz. The computer screen was placed approximately 80 cm in front of the participants’ eyes.

Auditory stimuli were two sinusoidal tones and an auditory response signal. The sinusoidal tones were delivered with 1000 or 800 Hz at 30 ms, 60 dB HL. The auditory response signal was a faint click at 500 Hz, 30 ms, 20 dB HL. All auditory stimuli were presented binaurally through earphones.

### Procedure

**FIGURE 1:**
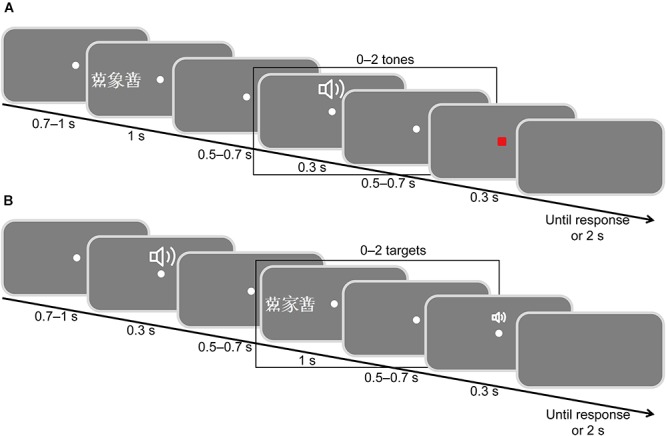
Schematic illustration of the stimulus display sequence. **(A)** Attending to visual stimuli while ignoring auditory stimuli. **(B)** Attending to auditory stimuli while ignoring visual stimuli. The Chinese character 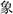 means elephant and 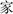 means home.

The experiment employed a cross-modal delayed response oddball paradigm (Figure 1; Wei et al., 2002; Chen et al., 2010). Participants were asked to fixate on the center of the screen and put on earphones. Each participant carried out two tasks. Task 1 involved attending to visual stimuli and ignoring auditory stimuli; Task 2 involved attending to auditory stimuli and ignoring visual stimuli. The orders of the two tasks (attending visual and attending auditory) were counterbalanced between participants. Both Task 1 and Task 2 included 800 trials. Participants were provided rest for 30 s after finishing 100 trials, and for 2 min after finishing a task. The experimental procedure was programmed with E-prime 1.1.

#### Task 1: Attending to Visual Stimuli While Ignoring Auditory Stimuli

Participants were instructed to attend to visual signals and ignore auditory signals (Figure 1A). They were required to fixate on the center point throughout the study and to view targets only utilizing peripheral vision. At the beginning of each trial, a white fixation dot was presented at the center of the screen for 500–700 ms. A target word and two flankers (flanker, target, flanker) were randomly presented for 1000 ms in either the left or right visual field on the horizontal meridian. The eccentricity of the target was 6°, and the spacing between the target and the flankers was at either 1° (crowded trials) or 4° (uncrowded trials). The crowded and uncrowded targets were presented randomly. According to a typical oddball paradigm (Hillyard et al., 1973), we set animal targets as deviant stimuli with a small probability (20%) and inanimate targets as standard stimuli with a large probability (80%). There were 160 animal targets, including 80 crowded and 80 uncrowded targets, and 640 inanimate targets, including 320 crowded and 320 uncrowded targets. Two flankers were selected randomly from the four pseudo-characters. After a randomized delay of 500–700 ms, 0–2 tones were presented. The duration of each tone was 30 ms. The time interval between the two tones was 500–700 ms. The frequency of 640 tones was 800 Hz, while 160 tones were 1000 Hz. Finally, a visual response signal (a small red square) was presented for 30 ms after a randomized inter-stimulus interval of 500–700 ms. Participants were required to judge the meanings of targets and to make responses by pressing one of the two mouse buttons with the thumb of either hand. Half of the participants were instructed to press the left mouse button if the meaning of target word was not an animal and to press the right mouse button if the target’s meaning was an animal, whereas the other half of the participants were instructed to perform the opposite action. Once the small red square appeared, participants were required to respond as quickly and accurately as possible. The next trial was presented once the participants had responded; the maximum time interval for response was 2000 ms.

#### Task 2: Attending to Auditory Stimuli While Ignoring Visual Stimuli

Participants were instructed to attend to auditory stimuli and ignore visual stimuli (Figure 1B). A tone (0–2 crowded or uncrowded words) and a faint click were presented successively. A randomized delay of 500–700 ms was inserted between the two stimuli. Participants were asked to discriminate the tone pitches and hold their response until the response signal (the faint click) was presented at the end of the trial. Half of the participants were instructed to press the left mouse button if the pitch was 800 Hz and to press the right mouse button if the pitch was 1000 Hz, while the other half of the participants were instructed to do the opposite. Other details of the task were the same as those reported in Task 1.

### Electrophysiological Recording

Continuous electroencephalogram (EEG) was acquired from Ag/AgCl electrodes mounted on a Quick-Cap (Neuroscan Inc.). Sixty-four electrodes were positioned according to the extended 10–20 system. All EEG electrodes were referenced to the left mastoid. The horizontal electrooculogram (EOG) was acquired using a bipolar pair of electrodes positioned at the external ocular canthi, and vertical EOGs were recorded from electrodes placed above and below the left eye. The EEG and EOG were digitized at 500 Hz with an amplifier bandpass of 0.05–100 Hz and were stored for offline analysis. All electrode impedances were maintained below 5 kΩ.

### EEG Analysis

EEGLAB (Delorme and Makeig, 2004) and MATLAB (The MathWorks, Inc., Massachusetts, United States) were used for offline EEG data processing. Continuous EEG data were re-referenced to the average of the right and left mastoids and were digitally low-pass filtered at 40 Hz. ERPs were time-locked to the onset of the target words, with an average epoch of 700 ms, including a 100 ms pre-stimulus baseline. All trials, no matter whether the response was correct or not, were included in analysis.

Ocular artifacts were rejected using a two-step procedure (Woodman and Luck, 2003; Luck, 2005). In the first step, for each point in the epoch, the mean value of the preceding 100 ms and that of the subsequent 100 ms were determined, and a difference value between two mean values was calculated. After this action was performed for each point, the largest difference value was compared with a threshold to determine whether the trial should be rejected. The single-trial waveforms were checked by visual inspection to determine a threshold value for each individual participant. Using the threshold, all clearly visible artifacts were rejected without the rejection of large numbers of artifact-free trials. We also excluded any participant for whom more than 25% of the trials were rejected owing to eye movements (Woodman and Luck, 2003). One participant’s data were excluded from the analysis because artifacts led the rejection of 46.8% of trials. On average, 11.4% of trials, ranging from 1.9 to 21.4% were rejected for the 17 remaining participants.

In the second step, the average horizontal EOG waveforms for left-target and right-target trials were calculated to assess the degree of residual eye movement activity. The average difference in voltage between left-target and right-target trials was less than 2.7 μV, which corresponded to an average eye movement of less than 0.2° (Lins et al., 1993; Zhang and Luck, 2009). Thus, it was determined that subjects were able to maintain fixation on the central fixation point throughout the task.

P1 (peaking at about 90 ms), N1 (about 160 ms), and P2 (about 230 ms) components were elicited by both crowded and uncrowded targets in both attended and unattended conditions (Figure 3). As shown in Figure 3, ERP component amplitude was measured from the mean amplitude of the 40-ms window centered at the grand average ERP peak latency and was separately determined for each condition (Näätänen et al., 2004).

The regions of interest (ROIs) were chosen according to previous studies and topographic information regarding P1, N1, P2, and crowded minus uncrowded wave differences observed in the current study (Figures 4, 5). Previous studies revealed the functional significance of the occipital region in crowding (Chen et al., 2014; Chicherov et al., 2014). The P1, N1, and P2 were predominantly distributed over the frontal, central, or occipital regions (Figure 4). As shown in Figure 5, results are consistent with previous studies that positive occipital distribution is accompanied by a negative frontal distribution (Clark and Hillyard, 1996; Flevaris et al., 2013). Thus, frontal and occipital electrodes were chosen as ROIs. ERP amplitudes at the F1, F2, F3, F4, F5, F6, and Fz electrode sites were averaged as measures of the frontal cluster, and those at the O1, O2, Oz, PO7, PO8, P7, and P8 electrode sites were averaged as measures of the occipital cluster (Zhang and Luck, 2009).

Planned comparisons were performed to address specific hypotheses. In order to assess whether crowding occurs automatically, each ERP component (P1, N1, and P2) was subjected to a paired samples *t*-test. These tests were conducted on the mean amplitude of ERP components to determine whether the means of the crowded and uncrowded conditions were equal. Paired *t*-tests were conducted in the attended and unattended conditions over both the frontal and occipital regions (four tests for each ERP component). To obtain a family wise confidence level of 0.95, a Bonferroni correction was used to adjust each individual confidence interval of 0.9875, and the corresponding significance level was set at 0.05/4 = 0.0125 (Armstrong, 2014).

In order to assess whether attention modulates crowding, difference amplitudes were obtained by subtracting the amplitudes of uncrowded ERP components from that of crowded ERP components in the attended and unattended conditions, respectively. For each ERP component, a paired sample *t*-test was conducted on the difference amplitudes to test whether the means of the attended and unattended conditions were equal. Paired *t*-tests were conducted over both the frontal and occipital region (two tests for each ERP component). The corresponding significance level was 0.05/2 = 0.025. Cohen’s *d* was used to estimate the effect size of the *t*-tests.

## Results

### Behavioral Data

**FIGURE 2:**
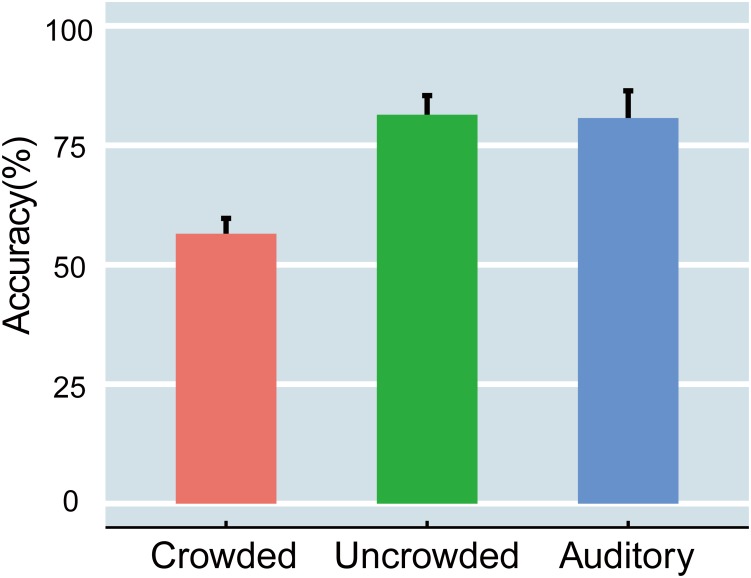
The accuracy of the crowded, uncrowded, and auditory tasks. The error bars indicate standard error.

Accuracy was computed for each participant in the crowded, uncrowded, and auditory conditions (Figure 2). A one-way, repeated measures analysis of variance (ANOVA) that was performed on accuracy scores revealed a significant main effect of condition [*F*(2,32) = 23.853, *p* < 0.001, η_p_^2^ = 0.599]. Specifically, accuracy was significantly lower in the crowded (ranging from 39 to 79%) than in the uncrowded condition (ranging from 53% to 96%) [*t*(16) = -7.396, *p* < 0.001, Cohen’s *d* = -1.794]. However, the accuracy difference between the uncrowded and auditory (ranging from 58 to 98%) conditions was not significant [*t*(16) = -0.144, *p* > 0.05, Cohen’s *d* = 0.035]. The results indicated that crowding led to a significant decline in performance, and that the identification of uncrowded targets had approximately the same level of difficulty as that observed in the auditory task.

### Event-Related Potential Data

**FIGURE 3:**
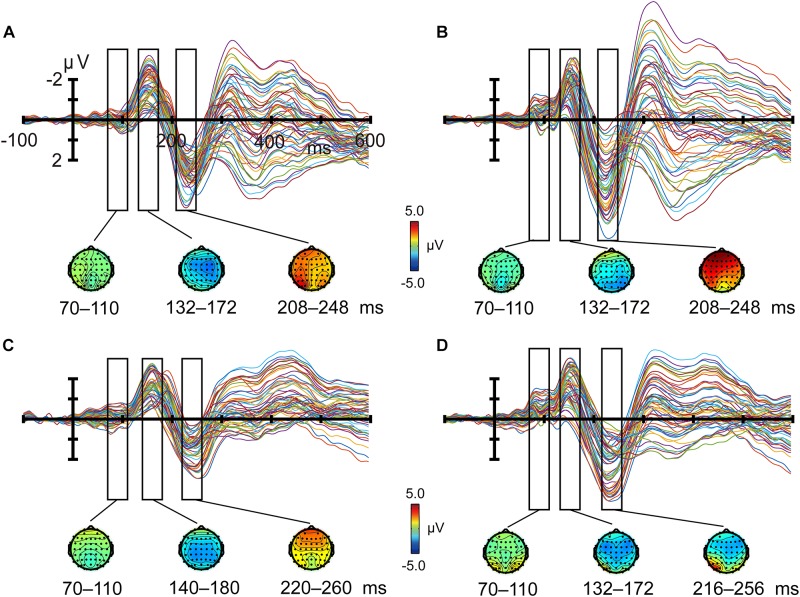
Butterfly plots of grand average event-related potentials and topographies. Crowded **(A)** and uncrowded **(B)** targets in the attended condition; crowded **(C)** and uncrowded **(D)** targets in the unattended condition.

**FIGURE 4:**
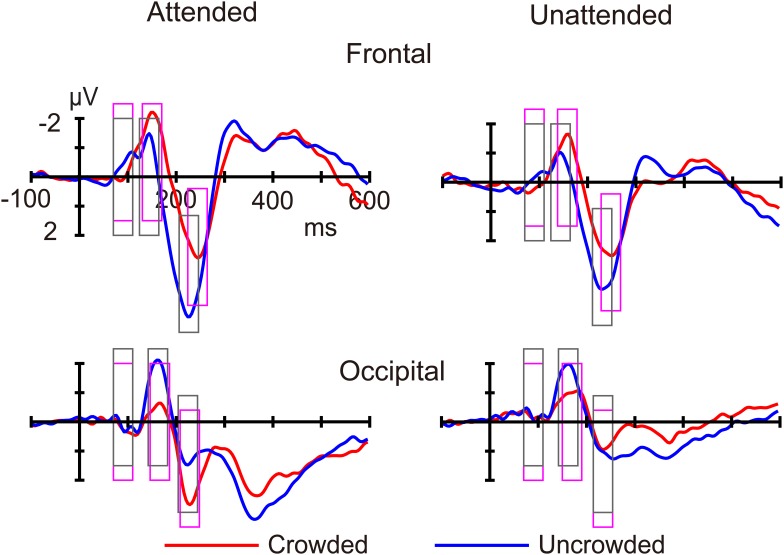
The average event-related potentials for crowded and uncrowded targets in the attended and unattended conditions. The analysis windows for crowded and uncrowded conditions were marked with magenta and black rectangles, respectively.

**FIGURE 5:**
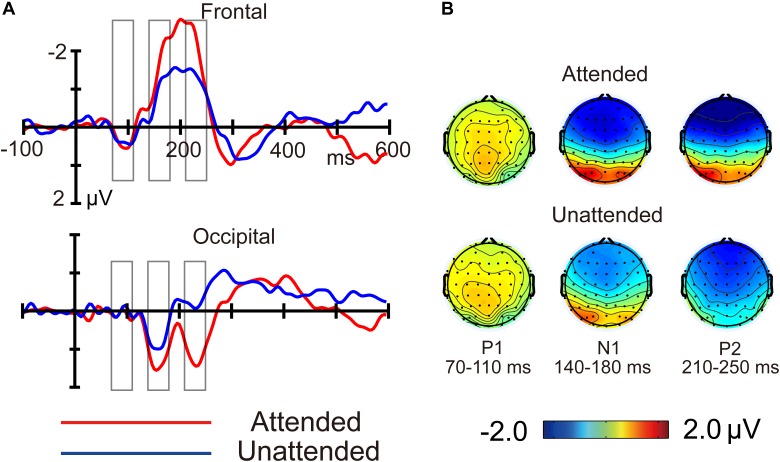
Difference in crowded minus uncrowded wave amplitudes **(A)** and topographies **(B)** in the attended and unattended conditions.

Figures 3, 4 shows ERP waveforms elicited by crowded and uncrowded targets in both attended and unattended conditions. An obvious separation between the crowded and uncrowded targets appeared during the N1–P2 stage (Figure 4). Figure 5A shows wave amplitude differences obtained by subtracting uncrowded ERPs from crowded ERPs in the attended and unattended conditions. Figure 5B shows the topographic results of the crowded minus uncrowded difference waves in the attended and unattended conditions during the P1, N1, and P2 stages. Compared with difference in amplitude wave in the unattended group, the difference in the amplitude wave of the attended group was more negative over the frontal region and more positive over the occipital region during the N1 and P2 stages (Figure 5).

**FIGURE 6:**
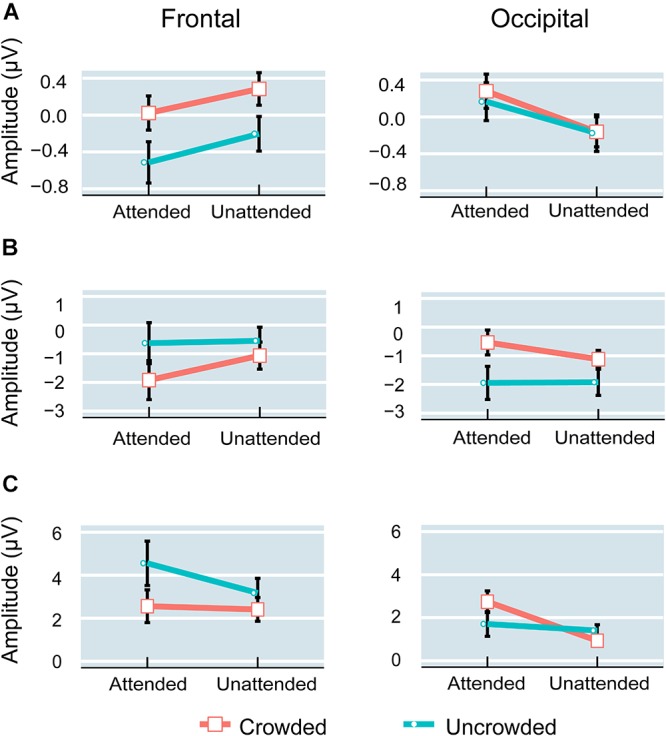
Amplitude of P1 **(A)**, N1 **(B)**, and P2 **(C)** components in the attended and unattended conditions over the frontal and occipital regions. Error bars indicate standard error.

Planned comparisons showed that crowded targets elicited a more positive P1 amplitudes compared with uncrowded targets in the attended condition [*t*(16) = 3.132, *p* < 0.01, Cohen’s *d* = 0.760] and in the unattended condition [*t*(16) = 4.094, *p* < 0.01, Cohen’s *d* = 0.993] over the frontal region. However, the difference between the crowded and uncrowded conditions was not significant in the attended and unattended conditions over the occipital region (*p*-values > 0.05; Figure 6A).

For the difference in wave amplitude (crowded – uncrowded) during the P1 stage, planned comparisons did not reveal any significant differences between the attended and unattended conditions over the frontal and occipital regions (*p*-values > 0.05).

Planned comparisons showed that crowded targets elicited a more negative N1 amplitude compared with uncrowded targets in the attended [*t*(16) = -4.829, *p* < 0.001, Cohen’s *d* = -1.171] and unattended [*t*(16) = -2.980, *p* < 0.01, Cohen’s *d* = -0.723] conditions over the frontal region. The crowded targets elicited a less negative N1 amplitude compared to uncrowded targets in both the attended [*t*(16) = 5.159, *p* < 0.001, Cohen’s *d* = 1.251] and unattended [*t*(16) = 3.394, *p* < 0.01, Cohen’s *d* = 0.823] conditions over the occipital region (Figure 6B).

During the N1 stage, planned comparisons revealed that the difference amplitude was more negative in the attended (-1.289 ± 0.267 μV) than in the unattended condition (-0.516 ± 0.173 μV) over the frontal region [*t*(16) = -2.852, *p* < 0.025, Cohen’s *d* = -0.692]. Further, the difference amplitude was more positive in the attended (1.410 ± 0.273 μV) than in the unattended condition (0.804 ± 0.237 μV) over the occipital region [*t*(16) = 2.665, *p* < 0.025, Cohen’s *d* = 0.646].

Planned comparisons showed that the crowded targets elicited less positive P2 amplitudes compared with the uncrowded targets in the attended [*t*(16) = -3.706, *p* < 0.01, Cohen’s *d* = -0.899] and unattended [*t*(16) = -3.461, *p* < 0.01, Cohen’s *d* = -0.839] conditions over the frontal region. Over the occipital region, the crowded targets elicited a more positive P2 amplitude as compared with the uncrowded targets in the attended condition [*t*(16) = 3.701, *p* < 0.01, Cohen’s *d* = 0.898], whereas there was no significant difference between the crowded and uncrowded targets in the unattended condition (*p* > 0.05; Figure 6C).

In the P2 stage, the difference amplitude was more negative in the attended (-1.996 ± 0.539 μV) than in the unattended condition (-0.7648 ± 0.221 μV) over the frontal region [*t*(16) = -2.829, *p* < 0.025, Cohen’s *d* = -0.686], whereas it was more positive in the attended (1.038 ± 0.280 μV) than in the unattended condition (-0.473 ± 0.240 μV) over the occipital region [*t*(16) = 5.255, *p* < 0.001, Cohen’s *d* = 1.275].

## Discussion

The present study combined a selective attention paradigm with a crowding paradigm to identify the relationship between attention and crowding. Consistent with previous studies (Pelli et al., 2004; Yeh et al., 2012; Peng et al., 2013), the ability to discriminate crowded targets dropped sharply compared to uncrowded targets (Figure 2). Additionally, the present study reproduced the N1 suppression in the crowding task (Chicherov et al., 2014; Ronconi et al., 2016). Furthermore, the current results suggest that crowding indeed occurs automatically and can be modulated by attention.

We found that crowded targets evoked a more positive P1 component compared to uncrowded targets, irrespective of whether they were attended or unattended. Previous studies have shown that the P1 wave reflects early visual processing of low-level characteristics of stimuli, such as luminance, intensity, eccentricity, and size (Johannes et al., 1995; Busch et al., 2004; Schadow et al., 2007). Chicherov et al. (2014) reported that the P1 amplitude positively correlated with the length of flankers in a Vernier crowding task. Consistent with the findings of such previous studies, P1 was found to reflect the early, lower-level visual processing of stimulus characteristics in the present study.

We found that the N1 was largest over the frontal–central region or occipital region (Figure 3), which is consistent with a previous study that a posterior N150 was distributed over the occipitoparietal region, and an anterior N155 was distributed over the frontal–central region (Di Russo et al., 2002). A posterior N1 component is usually accompanied by a smaller frontal component with reverse polarity (Clark and Hillyard, 1996). It may be in part due to volume transmission (Hedge et al., 2015). However, the entire frontal N1 is not due to volume transmission of N1 from occipital areas, but an overlap of a small positive component and a negative N155, thus we did not observe a frontal N1 component with reverse polarity in the current study. It is consistent with a notion that ERP events can be a complex result of underlying neural phenomena, which are difficult to study (Luck, 2014). Only one thing can be certain is that the timing of the ERP event in occipital and frontal areas (Luck, 2014).

Additionally, the current study found that crowded targets elicited a more negative frontal N1 and a less negative occipital N1 compared with uncrowded targets, irrespective of whether they were attended or unattended (Figure 4). These results replicated the findings of the occipital N1 suppression of crowding (Chicherov et al., 2014; Ronconi et al., 2016) and were consistent with the previous finding that crowding is associated with a suppression of V1, regardless of whether targets were attended or unattended (Millin et al., 2014). These results are in line with the bottom-up and pre-attentive account of crowding. Furthermore, the current study found that the difference in amplitude of the crowded minus uncrowded wave during the N1 stage was more negative over the frontal and more positive over the occipital region, when the targets were attended (Figures 5, 6). These results are in line with the top-down and attentional account of crowding. Thus, the current study provided electrophysiological evidence that crowding occurs automatically, and that it can be modulated by attention. The lateral inhibition and spatial pooling hypotheses predict that crowding occurs automatically (Westheimer and Hauske, 1975; Parkes et al., 2001), while the attentional resolution hypothesis predicts that crowding can be modulated by attention (He et al., 1996, 1997). However, these crowding hypotheses cannot fully predict the relationship between attention and crowding. The Gestalt grouping principle is more applicable to the current study. When the flankers and targets were closer, they were grouped, and therefore, crowding occurred. The Gestalt grouping hypothesis predicts that crowding is a combination of bottom-up (Müller et al., 2010; Marini and Marzi, 2016) and top-down (Wu et al., 2005; Khoe et al., 2006) processes. The current results most closely align with predictions made by the Gestalt grouping hypothesis of crowding.

In addition, the present findings of the P2 component are consistent with the Gestalt grouping hypothesis of crowding. Similar to the N1 component, the current study found that, crowded targets (compared to uncrowded targets) elicited a more negative frontal P2, irrespective of whether the targets were attended or unattended, as well as a less negative occipital P2 when targets were attended (Figure 4). Further, the difference in the crowded minus uncrowded wave amplitude during the P2 stage was more negative over the frontal and more positive over the occipital region when the targets were attended than when they were unattended (Figures 5, 6). It was also noted that the topographic elements of the difference waves were similar during the N1 and P2 stages (Figure 5B). A previous study reported that irrelevant probes superimposed on a moving image elicited an enhanced P2 component when the probes were contained within the boundaries of an object that was perceived as unitary, and that the topography of the P2 elicited by probes during object perception was distinct from that during fragment perception. These results indicate that the P2 wave is associated with grouping elements into unitary objects (Flevaris et al., 2013). Similar to the N1 component, P2 was found to be associated with Gestalt grouping in the present study.

The effects of target presentation time deserve further consideration. The presentation time of targets was 1 s in the present study, which was in line with the procedure followed in a previous study (Peng et al., 2013). Both the onset and offset of targets elicit neural activity (Baltzell and Billings, 2014). Using a long presentation time, we can avoid the overlapping of the neural activity of the offset with that of the cognitive process that we examined. However, more eye movement might be caused by visual stimuli with a longer presentation time. The current study rejected ocular artifacts using a two-step procedure (Woodman and Luck, 2003; Luck, 2005) and found that the remaining average eye movement was less than 0.2°. Thus, the effect of eye movement was excluded from ERP data. In addition, a longer presentation time may result in targets being able to access consciousness. The cross-modal delayed response of the oddball paradigm has been shown to control attention effectively; P300 component, an index of working memory and conscious perception (Salti et al., 2012), was only observed in the attended condition (Wei et al., 2002). This indicates that Wei et al.’s paradigm can exclude conscious access before and during the P300 stage in the unattended condition. The current study focused upon P1 (peaking at about 90 ms), N1 (about 160 ms), and P2 (about 230 ms) components. These components are earlier than P300, thus these components are not affected by consciousness in the unattended condition. Though we cannot exclude the possibility that the unattended targets access consciousness after the P300 stage, this does not affect the explanations of the findings on the P1, N1, and P2 components in the current study.

Finally, analytical methods used were checked to determine if they affected the conclusions of the current study. For instance, a two-step procedure to reject ocular artifacts (Woodman and Luck, 2003; Luck, 2005) was used. This procedure was utilized in a previous study on neural correlates of visual crowding (Chicherov et al., 2014). A separate study on the neural oscillatory correlates of crowding used Independent Component Analysis (ICA) to detect and correct ocular artifacts and removed epochs containing voltage deviation that exceeded ±75 μV (Ronconi et al., 2016). To the best of our knowledge, no previous research on this topic has checked whether the above two procedures are functionally equivalent. In addition, the current study use planned comparisons rather than ANOVA, because specific hypotheses that crowding occurs automatically and attention modulates crowding were maintained. It is necessary to determine whether similar statistical results can be obtained by using ANOVA. To address these issues, we conducted a supplementary analysis using ICA and ANOVA. Continuous EEG data were re-referenced, filtered, and segmented in the same manner described in Section 2.5 EEG analysis. Then, the ±75 μV ICA criterion was used to remove ocular artifacts. Similar ERP waveforms were obtained (Supplementary Figures S1–S3). Further, repeated measures ANOVA was conducted on amplitudes of P1, N1, and P2 waves. This analysis yielded similar statistical results (Supplementary Results). Therefore, the conclusions drawn in the current study do not appear to be affected by the analytical methods used.

## Conclusion

The present study employed a “cross-modal delayed response” oddball paradigm to investigate the relationship between attention and crowding. Previous findings that P1 reflects the early low-level processing of stimuli characteristics were replicated. We revealed that the N1 and P2 components were associated with the concept of Gestalt grouping in crowding. Specifically, crowding-related neural activity was found to appear, regardless of whether the crowded targets were attended or unattended. Additionally, neural activities appeared to be modulated by attention during the N1 and P2 stages. These results suggest that crowding occurs automatically and can be modulated by attention. Our results are consistent with previous studies on Gestalt grouping, which supports the notion that crowding originates from Gestalt grouping.

## Author Contributions

CP and YC designed the study. CP and CH performed the experiments. CP and YC decided on the final analyses and interpretation, and wrote the manuscript.

## Conflict of Interest Statement

The authors declare that the research was conducted in the absence of any commercial or financial relationships that could be construed as a potential conflict of interest.
