# Supplementary material for: The Temporal Dynamic Relationship Between Attention and Crowding: Electrophysiological Evidence From an Event-Related Potential Study
Source: Front Neurosci. 2018 Nov 22;12:844. doi: 10.3389/fnins.2018.00844 (PMC6261982; doi:10.3389/fnins.2018.00844)
Supplement: Supplementary file 1 [file Data_Sheet_1.pdf]

The Temporal Dynamic Relationship between Attention and Crowding: Electrophysiological Evidence from an Event-Related Potential Study

Chunhua Peng[1, 2], Chunmei Hu[1,2], Youguo Chen [3]*

[1] Laboratory of Emotion and Mental Health, Chongqing University of Arts and Sciences, Chongqing, China;
[2] Chongqing Collaborative Innovation Center for Brain Science, Chongqing, China;
[3] Key Laboratory of Cognition and Personality (Ministry of Education), Center of Studies for Psychology and Social Development, Faculty of Psychology, Southwest University, Chongqing, China.

***Correspondence:**
Youguo Chen
ygchen246@gmail.com

Supplementary Methods

Continuous EEG data were re-referenced, filtered and segmented just the same as descriptions in the formal text. Ocular artifacts were removed using the Independent Component Analysis (ICA) (Jung et al., 2000a, 2000b). Moreover, epochs containing voltage deviation that exceeds ±75 μV were also removed (Ronconi et al., 2016). On average, 1.54% of trials, ranging from 0.06% to 8.00% were rejected. No participant was excluded from the analysis.

The P1, N1, and P2 amplitudes were measured from the mean amplitude of the 40-ms window centered at the grand average ERP peak latency separately determined for each condition (Supplementary Figure 2). ERP amplitudes at the F1, F2, F3, F4, F5, F6, and Fz electrode sites were averaged as measures of the frontal cluster, and those at the O1, O2, Oz, PO7, PO8, P7, and P8 electrode sites were averaged as measures of the occipital cluster. A three-way repeated measures analysis of variance (ANOVA) was conducted on mean amplitudes of each component (P1, N1, and P2). ANOVA factors were attention (attended vs. unattended), crowding (crowded vs. uncrowded), and regions (frontal vs. occipital).

To further assess whether attention modulates crowding, difference amplitudes were obtained by subtracting the amplitudes of uncrowded ERP components from that of crowded ERP components in the attended and unattended conditions, respectively. If crowding × attention or/and crowding × attention × regions interactions were significant, a two-way repeated measures ANOVA was conducted on difference amplitudes during each component stage. ANOVA factors were attention (attended vs. unattended) and regions (frontal vs. occipital). A Greenhouse-Geisser correction was used to correct for any violations of sphericity (Greenhouse and Geisser, 1959), and the partial eta squared (η_p^2) was used to estimate the ANOVA effect size (Levine and Hullett, 2002).

Supplementary Results

Behavioral results. The accuracy was computed for each participant in the crowded, uncrowded, and auditory conditions. A one-way repeated measures ANOVA performed on the accuracy revealed a significant main effect of conditions [$F(2, 34) = 26.533$, $p < 0.001$, $\eta_p^2 = 0.609$]. The accuracy was significant lower in the crowded (0.565 ± 0.030 μV) than in the uncrowded condition (0.811 ± 0.037 μV) [$t(17) = -7.760$, $p < 0.001$, Cohen's $d = -1.829$], and the difference of accuracy between uncrowded and auditory (0.807 ± 0.054 μV) conditions was not significant [$t(17) = 0.105$, $p > 0.05$, Cohen's $d = 0.025$]. The results indicated that crowding declined performance significantly; the identification of uncrowded targets had the same level of difficulty as in the auditory task.

EEG results. Supplementary Figure S1 and S2 shows ERP waveforms elicited by

crowded and uncrowded targets in both attended and unattended conditions. A repeated measures ANOVA conducted on the P1 amplitude found a significant main effect of crowding [$F(1, 17) = 5.313$, $p < 0.05$, $\eta_p^2 = 0.238$] and a significant interaction of attention × regions [$F(1, 17) = 17.539$, $p < 0.01$, $\eta_p^2 = 0.508$]. The other main effects and interactions were not significant (p values > 0.05). Crowded targets evoked a more positive P1 component (−0.119 ± 0.118 μV) compared with uncrowded targets (−0.321 ± 0.112 μV). A simple effects analysis on attention × regions interaction revealed that the difference of P1 amplitude betweeen attended and unattended conditions was not significant over the frontal region ($p > 0.05$), whereas the P1 amplitude was more positive in the attended condition (0.149 ± 0.187 μV) than that in the unattended condition (-0.306 ± 0.166 μV) over the occipital region [$F(1, 17) = 18.354$, $p < 0.01$, $\eta_\mathrm{p}^2 = 0.519$; Supplementary Figure S3A].

For N1 peak amplitude, a repeated measures ANOVA revealed significant interactions of attention × regions [$F(1,17) = 4.728$, $p < 0.05$, $\eta_p^2 = 0.218$], crowding × regions [$F(1,17) = 50.982$, $p < 0.001$, $\eta_p^2 = 0.750$] and crowding × attention × regions [$F(1,17) = 43.623$, $p < 0.001$, $\eta_p^2 = 0.720$]. The other main effects and interactions were not significant (p values > 0.05). A simple effects analysis on crowding × attention × regions interaction revealed that the crowded targets elicited a more negative N1 amplitude compared with the uncrowded targets in the attended [$F(1, 17) = 24.421$, $p < 0.001$, $\eta_\mathrm{p}^2 = 0.590$] and unattended [$F(1, 17) = 12.281$, $p < 0.01$, $\eta_\mathrm{p}^2 = 0.419$] conditions over the frontal region. The crowded targets elicited a less negative N1 amplitude compared with the uncrowded targets in the attended [$F(1, 17) = 34.114$, $p < 0.01$, $\eta_\mathrm{p}^2 = 0.667$] and unattended [$F(1, 17) = 10.878$, $p < 0.01$, $\eta_\mathrm{p}^2 = 0.390$] conditions over the occipital region (Supplementary Figure S3B).

A repeated measures ANOVA conducted on difference amplitude during N1 stage revealed a significant main effect of regions [$F(1, 17) = 50.982$, $p < 0.001$, $\eta_\mathrm{p}^2 = 0.750$] and a significant interaction of attention × regions [$F(1, 17) = 43.623$, $p < 0.001$, $\eta_\mathrm{p}^2 = 0.720$]. The main effect of attention was not significant ($p > 0.05$). A simple effects analysis on attention × regions interaction revealed that the difference amplitude was more negative in the attended (-1.252 ± 0.253 μV) than in the unattended condition (-0.494 ± 0.141 μV) over the frontal region [$F(1, 17) = 11.512$, $p < 0.01$, $\eta_\mathrm{p}^2 = 0.404$]. Further, the difference amplitude was more positive in the attended (1.463 ± 0.251 μV) than in the unattended condition (0.686 ± 0.208 μV) over the occipital region [$F(1, 17) = 15.037$, $p < 0.01$, $\eta_\mathrm{p}^2 = 0.469$].

For P2 amplitude, repeated measures ANOVA revealed a significant main effect of crowding [$F(1, 17) = 8.273$, $p < 0.05$, $\eta_p^2 = 0.327$] and significant interactions of crowding × regions [$F(1,17) = 18.020$, $p < 0.01$, $\eta_p^2 = 0.515$] and crowding × attention × regions [$F(1,17) = 26.757$, $p < 0.001$, $\eta_p^2 = 0.611$]. The other main effects and interactions were not significant (p values > 0.05). A simple effects analysis on crowding × attention × regions interaction revealed that the crowded targets elicited less positive P2 amplitudes compared with the uncrowded targets in the attended [$F(1,17) = 19.180$, $p < 0.001$, $\eta_p^2 = 0.530$] and unattended [$F(1,17) = 14.283$, $p < 0.01$, $\eta_p^2 = 0.457$] conditions over the frontal region. Over the occipital region, the crowded

targets elicited a more positive P2 amplitude as compared with the uncrowded targets in the attended condition [$F(1,17) = 15.189$, $p < 0.01$, $\eta_p^2 = 0.472$], whereas there was no significant difference between the crowded and uncrowded targets in the unattended condition ($p > 0.05$; Supplementary Figure S3C).

A repeated measures ANOVA conducted on difference amplitude during the P2 stage revealed a significant main effect of regions [$F(1, 17) = 18.020$, $p < 0.01$, $\eta_p^2 = 0.515$] and a significant attention × regions interaction [$F(1, 17) = 26.757$, $p < 0.001$, $\eta_p^2 = 0.611$]. The main effect of attention was not significant ($p > 0.05$). A simple effects analysis on attention × regions interaction revealed that the difference amplitude was more negative in the attended (-2.129 ± 0.486 μV) than in the unattended condition (-1.041 ± 0.275 μV) over the frontal region [$F(1, 17) = 7.213$, $p < 0.05$, $\eta_p^2 = 0.298$], whereas it was more positive in the attended (1.251 ± 0.321 μV) than in the unattended condition (-0.135 ± 0.279 μV) over the occipital region [$F(1, 17) = 33.210$, $p < 0.001$, $\eta_p^2 = 0.661$].

Supplementary References

Greenhouse, S. W., and Geisser, S. (1959). On methods in the analysis of profile data. Psychometrika 24, 95–112. doi:10.1007/BF02289823.

Jung, T. P., Makeig, S., Humphries, C., Lee, T. W., McKeown, M. J., Iragui, V., et al. (2000a). Removing electroencephalographic artifacts by blind source separation. Psychophysiology 37, 163–78. doi:10.1111/1469-8986.3720163.

Jung, T. P., Makeig, S., Westerfield, M., Townsend, J., Courchesne, E., and Sejnowski, T. J. (2000b). Removal of eye activity artifacts from visual event-related potentials in normal and clinical subjects. Clin Neurophysiol 111, 1745–58. doi:10.1016/S1388-2457(00)00386-2.

Levine, T. R., and Hullett, C. R. (2002). Eta squared, partial eta squared, and misreporting of effect size in communication research. Hum. Commun. Res. 28, 612–625. doi: 10.1111/j.1468-2958.2002.tb00828.x.

Ronconi, L., Bertoni, S., and Bellacosa Marotti, R. (2016). The neural origins of visual crowding as revealed by event-related potentials and oscillatory dynamics. Cortex 79, 87–98. doi:10.1016/j.cortex.2016.03.005.

Figure Captions

Supplementary Figure S1. Butterfly plots of grand average event-related potentials and topographies. Crowded (A) and uncrowded (B) targets in the attended condition; crowded (C) and uncrowded (D) targets in the unattended condition.

Supplementary Figure S2. The average event-related potentials for crowded and uncrowded targets in the attended and unattended conditions. The analysis windows for crowded and uncrowded conditions were marked with magenta and black rectangles respectively.

Supplementary Figure S3. Amplitude of P1 (A), N1 (B), and P2 (C) components in the attended and unattended conditions over the frontal and occipital regions. Error bars indicate standard error.
