# Supplementary figures and images for: The Temporal Dynamic Relationship Between Attention and Crowding: Electrophysiological Evidence From an Event-Related Potential Study
Source: Front Neurosci. 2018 Nov 22;12:844. doi: 10.3389/fnins.2018.00844 (PMC6261982; doi:10.3389/fnins.2018.00844)

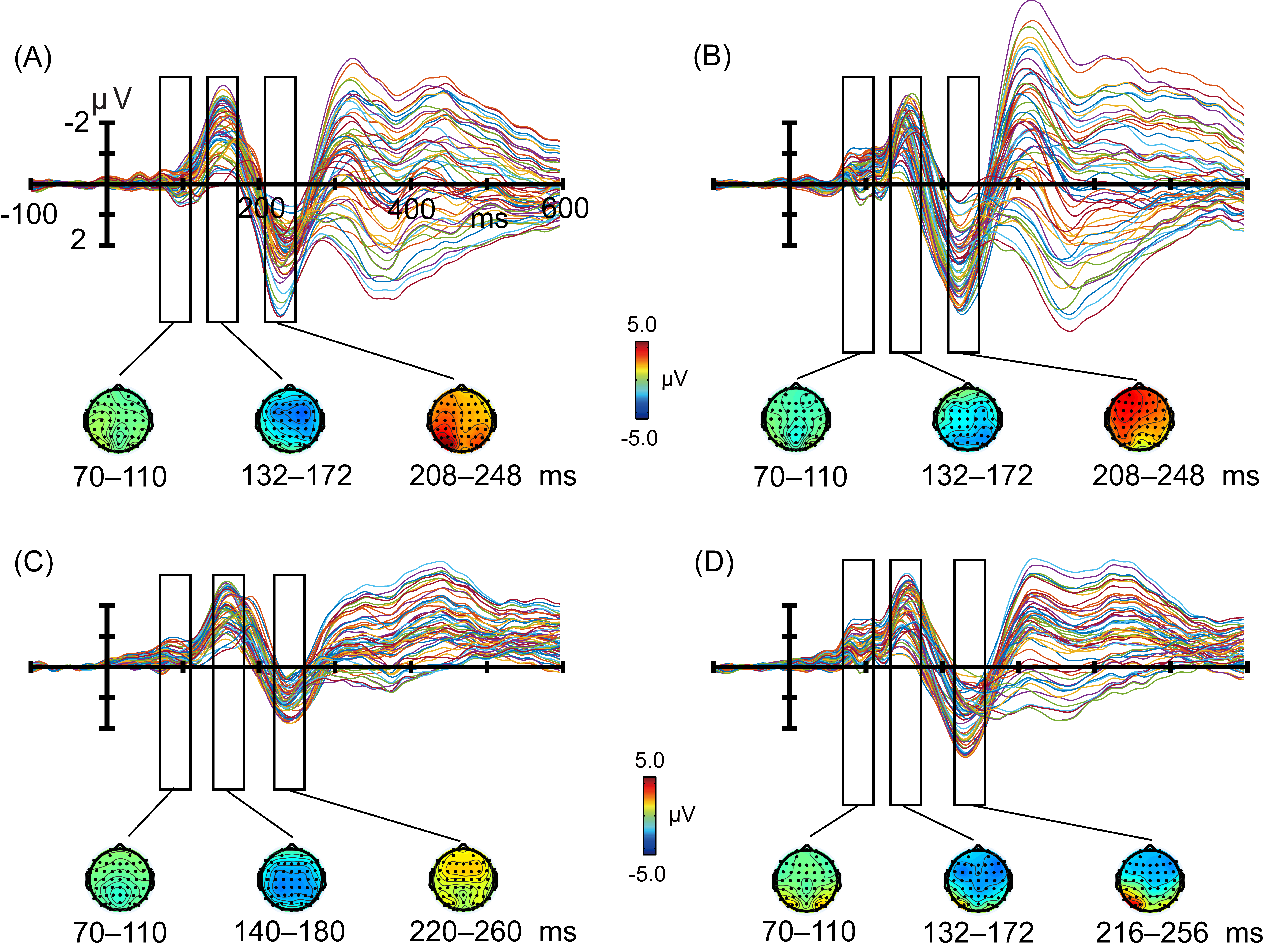

Supplement: Supplementary file 2 [file Image_1.TIF]

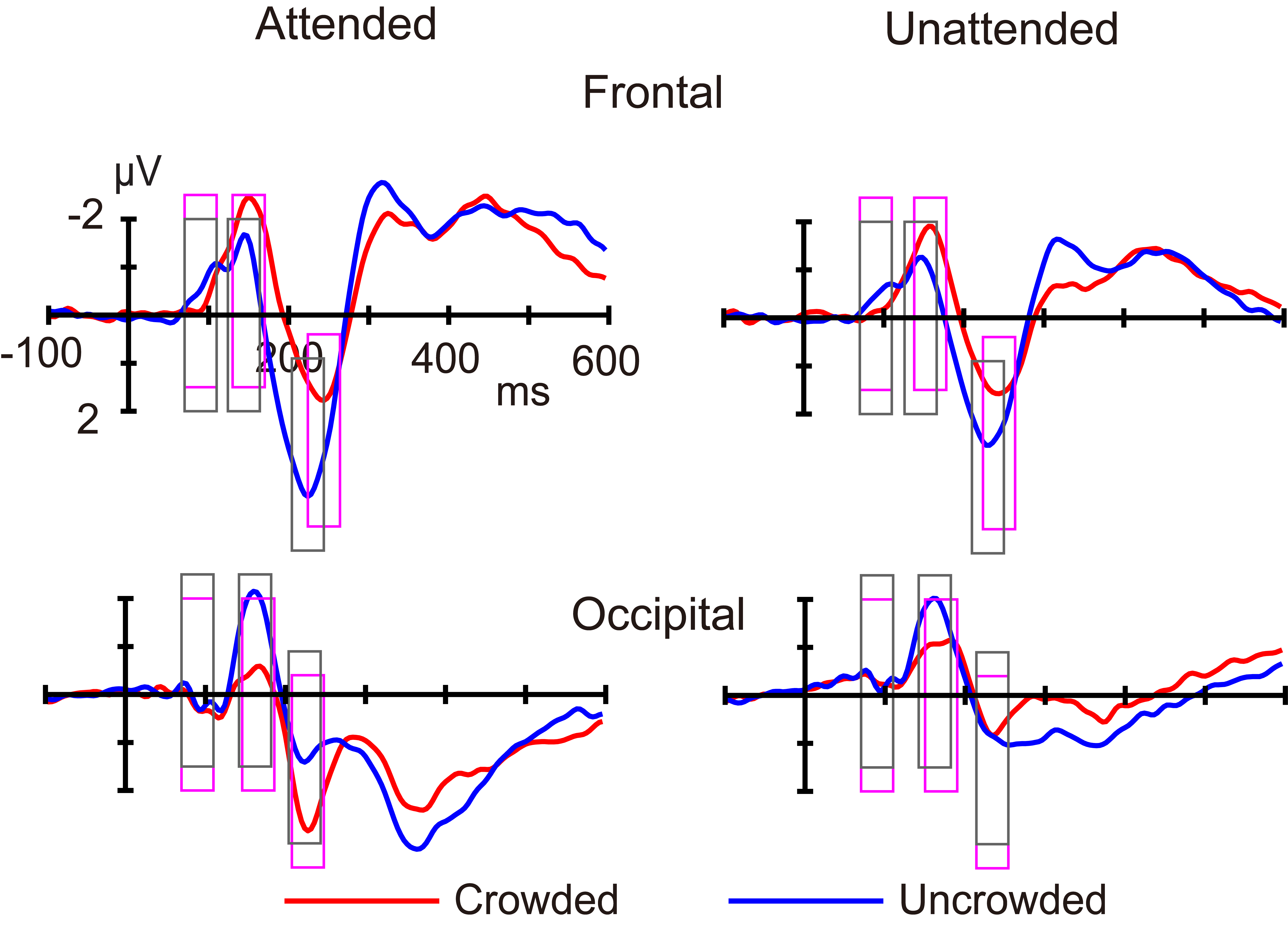

Supplement: Supplementary file 3 [file Image_2.TIF]

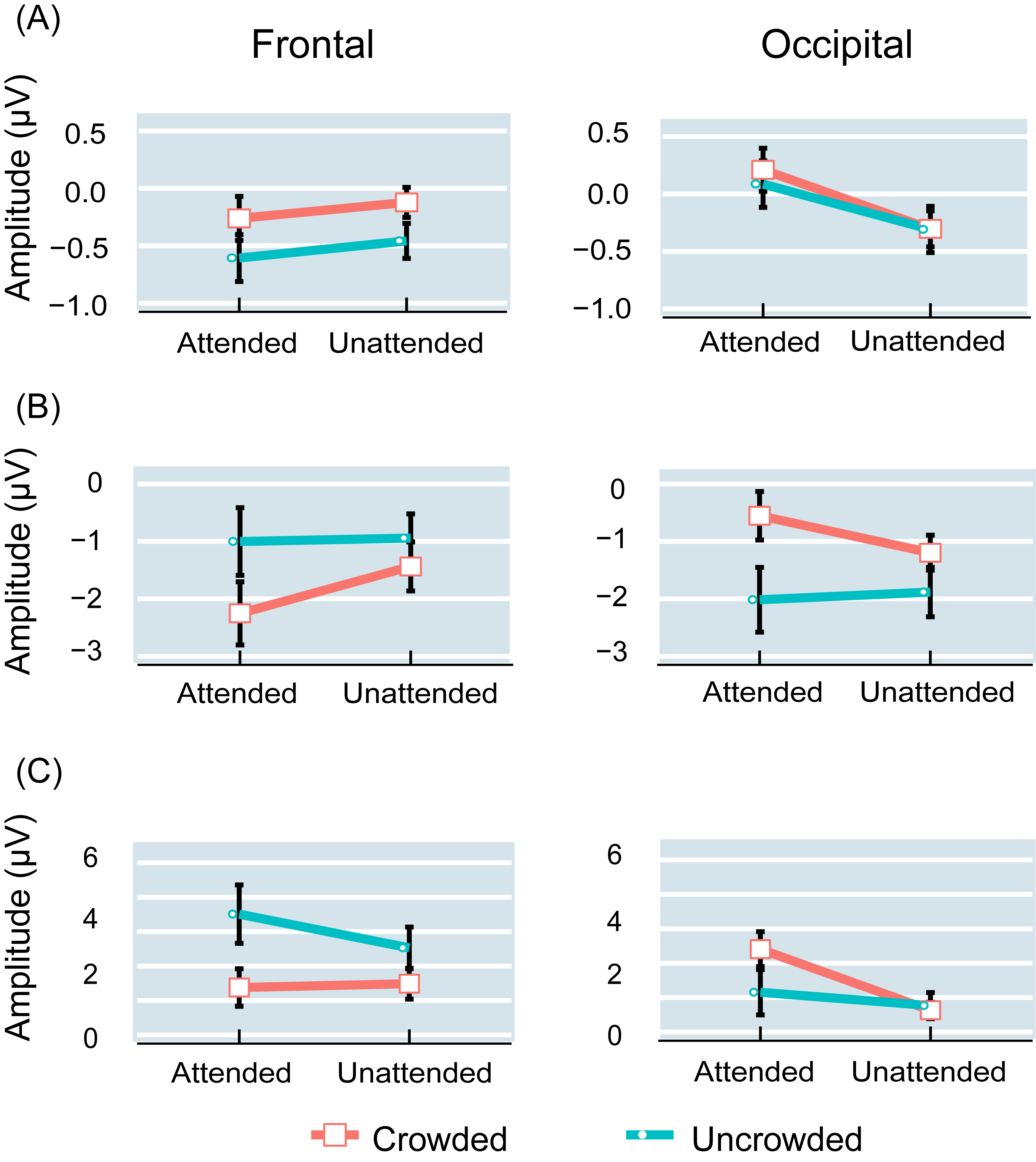

Supplement: Supplementary file 4 [file Image_3.TIF]
